# Deficiency of salt‐inducible kinase 2 (SIK2) promotes immune injury by inhibiting the maturation of lymphocytes

**DOI:** 10.1002/mco2.366

**Published:** 2023-09-11

**Authors:** Jiaojiao Zhu, Chao Li, Ping Wang, Yuhao Liu, Zhongqiu Li, Zhongmin Chen, Ying Zhang, Bin Wang, Xueping Li, Ziyan Yan, Xinxin Liang, Shenghui Zhou, Xingkun Ao, Maoxiang Zhu, Pingkun Zhou, Yongqing Gu

**Affiliations:** ^1^ Beijing Key Laboratory for Radiobiology Beijing Institute of Radiation Medicine Beijing P. R. China; ^2^ School of Life Science Shihezi University, Shihezi Xinjiang Province P. R. China; ^3^ Medical School Shihezi University, Shihezi Xinjiang Province P. R. China; ^4^ PLA Rocket Force Characteristic Medical Center Beijing P. R. China; ^5^ Hengyang Medical College University of South China Hengyang Hunan Province P. R. China

**Keywords:** immune injury, lymphocytes, maturation, SIK2

## Abstract

Salt‐inducible kinase 2 (SIK2) belongs to the serine/threonine protein kinases of the AMPK/SNF1 family, which has important roles in cell cycle, tumor, melanogenesis, neuronal damage repair and apoptosis. Recent studies showed that SIK2 regulates the macrophage polarization to make a balance between inflammation and macrophage. Macrophage is critical to initiate immune regulation, however, whether SIK2 can be involved in immune regulation is not still well understood. Here, we revealed that the protein of SIK2 was highly expressed in thymus, spleen, lung, and brain. And SIK2 protein content increased in RAW264.7 and AHH1 cells with a time and dose‐dependent after‐ionizing radiation (IR). Inhibition of SIK2 could promote AHH1 cells apoptosis Moreover, we used the Cre‐LoxP system to construct the SIK2^+/−^ mice, and the research on function suggested that the deficiency of SIK2 could promote the sensitivity of IR. The deficiency of SIK2 promoted the immune injury via inhibiting the maturation of T cells and B cells. Furthermore, the TCRβ rearrangement was inhibited by the deficiency of SIK2. Collectively, this study demonstrated that SIK2 provides an essential function of regulating immune injury, which will provide new ideas for the treatment of immune injury‐related diseases.

## 1 INTRODUCTION

Salt‐inducible kinase (SIK) belongs to the serine/threonine protein kinases of the AMPK/SNF1 family, including SIK1, SIK2, and SIK3.^1^, ^2^ SIK1 highly expresses in the adrenal gland, reduces the expression of corticotropin induced steroid genes and also maintains the dynamic balance of liver glucose metabolism and myocyte survival.^3^, ^4^, ^5^, ^6^, ^7^, ^8^ SIK3 can be associated with the regulation of proliferation, migration, inflammation, apoptosis, and arterial restenosis.^9^, ^10^, ^11^ What is more, SIK1 and SIK3 play an important role in immune regulation.^12^, ^13^ SIK2 widely expresses in human and mouse adipose tissues, which mainly participates in the regulation of glucose and lipid metabolism through phosphorylation.^14^, ^15^, ^16^, ^17^ Furthermore, SIK2 has important roles in cell cycle, tumor, melanogenesis, neuronal damage repair, and apoptosis.^18^, ^19^, ^20^, ^21^ Recent studies showed that SIK2 regulates the macrophage polarization to make a balance between inflammation and macrophage,^9^, ^22^ given the function of macrophage, which indicate that SIK2 might be involved in the regulation of immune response.

In our previous study, the protein content of SIK2 was increased under ionizing radiation (IR), and SIK2 interacted with the radiation‐responsive protein DNA‐dependent protein kinase catalytic subunits (DNA‐PKcs). A large number of studies suggested that DNA‐PKcs is a key protein in DNA damage repair and mitotic processes, and the lymphocyte maturation and the V(D)J recombination process were impaired in DNA‐PKcs^−/−^ mice.^1^, ^23^, ^24^, ^25^ These data indicated that SIK2 can be involved in the IR‐induced immune injury response. Immune system is a radiation‐sensitive tissue in the body. Many studies have confirmed that ionizing radiation (IR) can induce immune damage in the system.^26^, ^27^, ^28^ Localized 8.7 cGy over 10 days resulted in longstanding systemic lymphoid hypoplasia and low dose radiation could induce immune suppression.^29^, ^30^ However, the mechanism is still unclear, and it is important to find a target that can evaluate the application value of radiation‐induced immune injury.

In this study, we provide ample evidence that SIK2 served as a critical protein in immune injury. We found that SIK2 expression was significantly upregulated in a time and dose‐dependent manner in RAW264.7 cells with IR stimulation. By constructing the SIK2^+/–^ mice, functional studies demonstrated that SIK2^+/−^ had an effect on organism development and facilitated the sensitivity of IR. In addition, we demonstrated that deficiency of SIK2 expression significantly inhibits the maturation of T cells and B cells to impair immunity. Together, we provide new insights of SIK2 function and the potential application to immune injury.

## 2 RESULTS

### 2.1 Protein content of SIK2 increases after ionizing radiation

**FIGURE 1:**
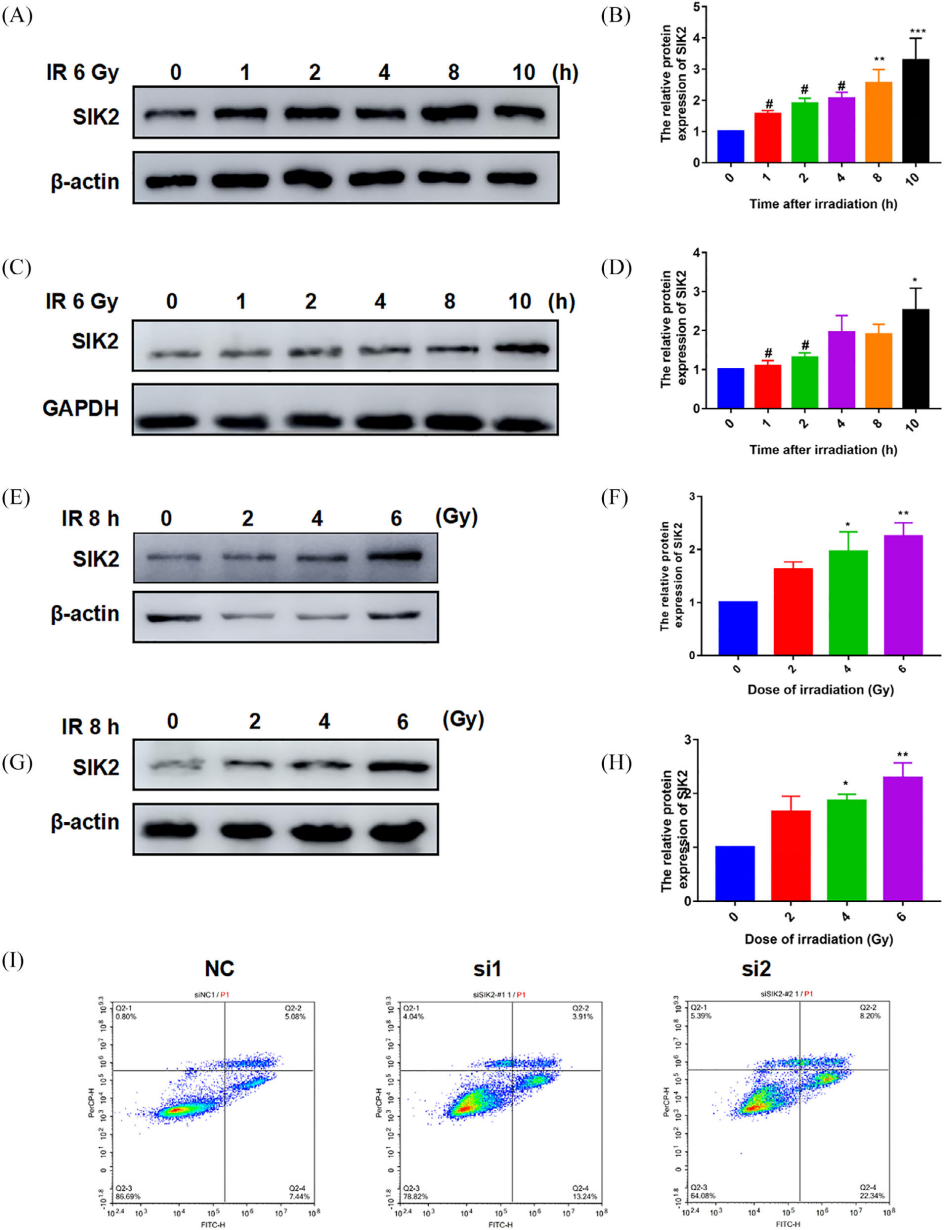
Protein content of SIK2 increases after ionizing radiation. (A) The SIK2 protein analysis with different radiation time stimulation in RAW264.7. (B) Quantified results of A. (C) The SIK2 protein analysis with different radiation time stimulation in AHH1. (D) Quantified results of C. (E) The SIK2 protein analysis with different radiation dose stimulation in RAW264.7. (F) Quantified results of E. (G) The SIK2 protein analysis with different radiation dose stimulation in AHH1. (H) Quantified results of G. (I) Inhibited of SIK2, AHH1 cells apoptosis was detected by flow cytometry. Data are mean as—x— ± *SD*, *p* values were determined by *t*‐test, “*” means compared with “0” group, “#” means compared with “10” group, ^*^ *p* < 0.05, ^**^ *p* < 0.01, ^***^ *p* < 0.001 and ^#^ *p* < 0.05 mean the statistical difference significantly.

Findings from our group have revealed that SIK2 can interact with the radiation response protein DNA‐PKcs. Moreover, SIK2 can regulate signal transduction, cell cycle, tumor formation, melanin production, neuronal injury repair, autophagy, and apoptosis. However, whether SIK2 has a response on radiation is poorly understood. To better determine the function of SIK2, bioinformatic analysis of the SIK2 gene sequences using MEGA version 6.0 software revealed that the evolution of the SIK2 gene is highly conserved in the genomes of human, rabbit, mouse, rat, and domestic chicken (Figure S1A). Furthermore, it was shown that the SIK2 amino‐acid sequence analyzed by DNAMAN is similarity to human, rabbit, mouse, rat, and domestic chicken (Figure S1B). These data suggested that SIK2 might play an important role in organism development. Therefore, the expression profile of SIK2 protein analysis found that SIK2 was widely expression in many tissues and highly expressed in brain, lung, thymus, testis, and spleen (Figure S1C). Many researches have shown that IR usually induces immune system injury, brain injury, lung injury, and reproductive system injury. These tissue injury induced by IR are consistent with SIK2 expression profiles. It was found that the protein content of SIK2 in RAW264.7 and AHH1 cells increased in a time dependent manner with the extension of IR stimulation time (Figure 1A‐D). Moreover, the protein expression of SIK2 increased dose‐dependently with the increase of IR dose (Figure 1E‐H). What is more, inhibition SIK2 in AHH1 cells could increase apoptosis of AHH1 cells (Figure 1I), suggesting SIK2 plays a role in cell development. Together, these data indicated that SIK2 has a radiation‐responsive function and may be involved in tissue injury induced by IR.

### 2.2 Construction and identification of SIK2^+/−^ mice model

**FIGURE 2:**
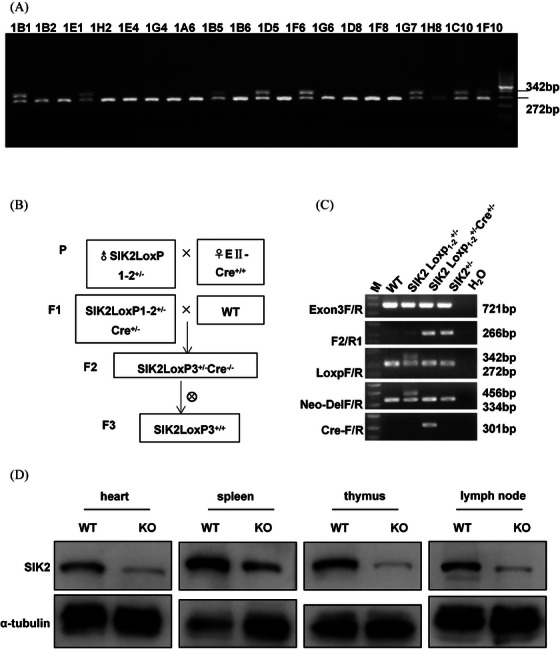
Construction and identification of SIK2^+/−^ mice model. (A) The identification of positive site by PCR. (B) The breeding process. (C) Identifying the gene type by PCR. (D) Western blot analyzed the expression of SIK2 in heart, spleen, thymus and lymph node.

Since SIK2 was found to be highly expressed in immune system tissues and cells and had a radiation‐responsive, we next generated SIK2 knockout mice by using Cre‐LoxP system to analyze the function of SIK2 in immune injury and IR response. According to the overall targeting strategy, the ES cell targeting vector pSIK2‐KO with two LoxP sites was constructed, and two LoxP sites were integrated into the intron flanking exon 2 and 3 of the SIK2 gene by homologous recombination (Figure S2A). We selected positive ES clones with two LoxP sites by extracting ES genomic DNA after culture selection, and identified the size of the 3′‐end long chain PCR product was 4.3 kb (Figure S2B), and positive LoxP site and WT can be detected at 342 and 272 bp bands, respectively (Figure 2A). In line with expectations, it was suggested that the two LoxP sites were successfully inserted into the intron on both sides of the SIK2 exon 2 and 3.

In addition, we synthesized probes using Neo probe‐F/R as probe primers to hybridize with genomic DNA fragments from the screened positive ES cells. Southern blot results showed that the DNA fragment containing the 5′‐homology arm was 18.8 kb and that containing the 3′‐homology arm was 7.2 kb (Figure S2C). Amplified with NGH001 Neo_Del F/R primer, the PCR genotyping results showing WT allele can be detected at 334 bp and LoxP1 site can be detected at 456 bp (Figure S2D), indicating that Neo locus was successfully removed after mating with FLP females. Amplified with NGH001‐LoxP‐F/R primer, WT allele can be detected at 272 and a 342 bp allele was inserted at LoxP2 locus (Figure 2A). These data indicated LoxP site was successfully inserted.

SIK2 LoxP1‐2^+/–^ chimera mice were hybridized with E‐Cre^+/+^ tool mice after purification to obtain SIK2LoxP1‐2^+/–^ Cre^+/−^ mice. Then, the SIK2LoxP3^+/−^Cre^+/–^ mice were hybridized with the WT mice to obtain SIK2LoxP_3_^+/−^Cre^−/−^ mice. Next, the male and female SIK2LoxP_3_^+/−^Cre^−/−^ mice hybridized to obtain SIK2^−/−^, and the result showed that mice with genotype SIK2^−/−^ were not successfully constructed (Figure 2B). Both sequencing and PCR analysis showed that SIK2 was successfully knockout (Figure 2C). Moreover, the protein of SIK2 decreased in heart, spleen, thymus and lymph node in SIK2^+/–^ mice, suggesting the efficiency of SIK2 knockout was in a good condition and SIK2^+/–^ mice can be used to perform our research (Figure 2D).

### 2.3 The deficiency of SIK2 has an effect on organism development and increases the sensitivity of IR

**FIGURE 3:**
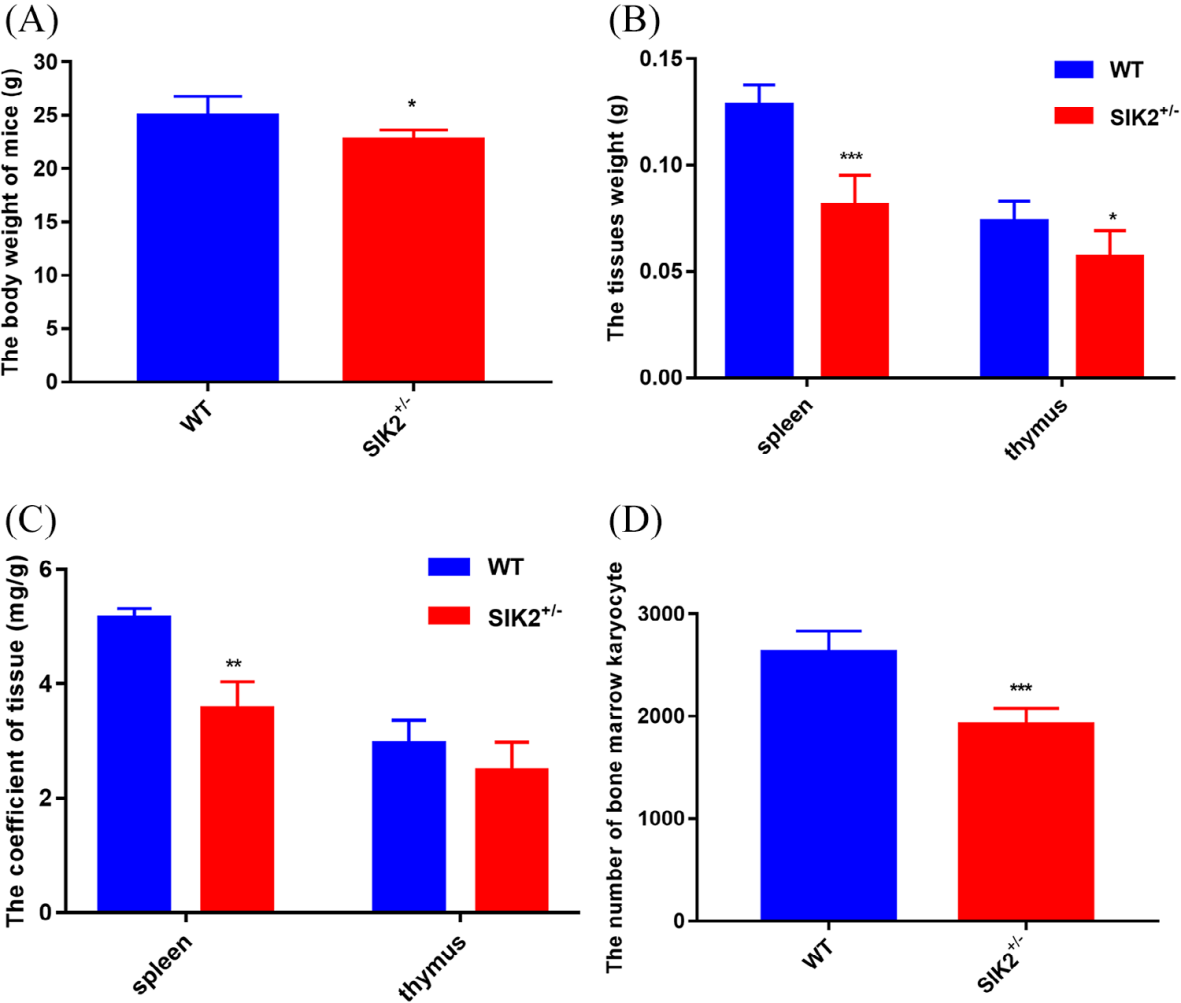
The deficiency of SIK2 has an effect on organism development and increases the sensitivity of IR. (A) The effect on body weight of the deficiency of SIK2 in mice. (B) The tissue weight of spleen and thymus in mice. (C) The tissue coefficient of spleen and thymus in mice. (D) The number of karyocyte in bone marrow. Dose of IR is 8 Gy. Data are mean as —x— ± *SD*, *p* values were determined by *t*‐test, and ^*^ *p* < 0.05, ^**^ *p* < 0.01, and ^**^ *p* < 0.001 mean the statistical difference significantly.

The data presented earlier from cell models suggested that SIK2 had a response on immune cells with IR treatment, which indicated that SIK2 might play an important in regulation of immune injury. To directly test this hypothesis, we next explored whether the deficiency of SIK2 can alter the IR‐induced response. Compared with WT mice, the body weight of SIK2^+/−^ mice significantly decreased (Figure 3A), and the weight and coefficient of thymus and spleen also reduced (Figure 3B and C). Moreover, the bone marrow karyocyte reduced in SIK2^+/−^ mice (Figure 3D), suggesting the deficiency of SIK2 has effect on the organism development. Furthermore, blood routine examination showed that the number of white blood cells (WBC), red blood cells (RBC), and platelet (PLT) decreased in SIK2^+/−^ mice blood, and the hemoglobin (HGB) concentration was significantly reduced (Table 1), indicating the deficiency of SIK2 might involve in regulation of immune cells. These data indicated that SIK2 is critical for regulation of the organism development and sensitivity of IR.

**TABLE 1:** Comparison of blood biochemical parameters between WT and SIK2^+/–^ mice.

Index	WT	SIK2^+/−^
Total number of cases	7	7
WBC (×10^9^/L)	6.4 ± 0.6	5.5 ± 0.5^*^
RBC (×10^12^/L)	7.6 ± 0.53	6.15 ± 0.58^**^
PLT (×10^9^/L)	449.0 ± 7.7	411.2 ± 6.4^*^
HGB (g/L)	101.3 ± 7.7	91.5 ± 2.9^*^

*Note*: Values are given as the mean ± SD.

^*^ *p* < 0.05 and ^**^ *p* < 0.01 compared with WT group, the difference was statistically significant.

WT: wild‐type group; SIK2^+/−^: the deficiency of SIK2 group; WBC: white blood cells; RBC: red blood cells; PLT: Platelet; HGB: hemoglobin.

### 2.4 The deficiency of SIK2 promotes the immune injury

**FIGURE 4:**
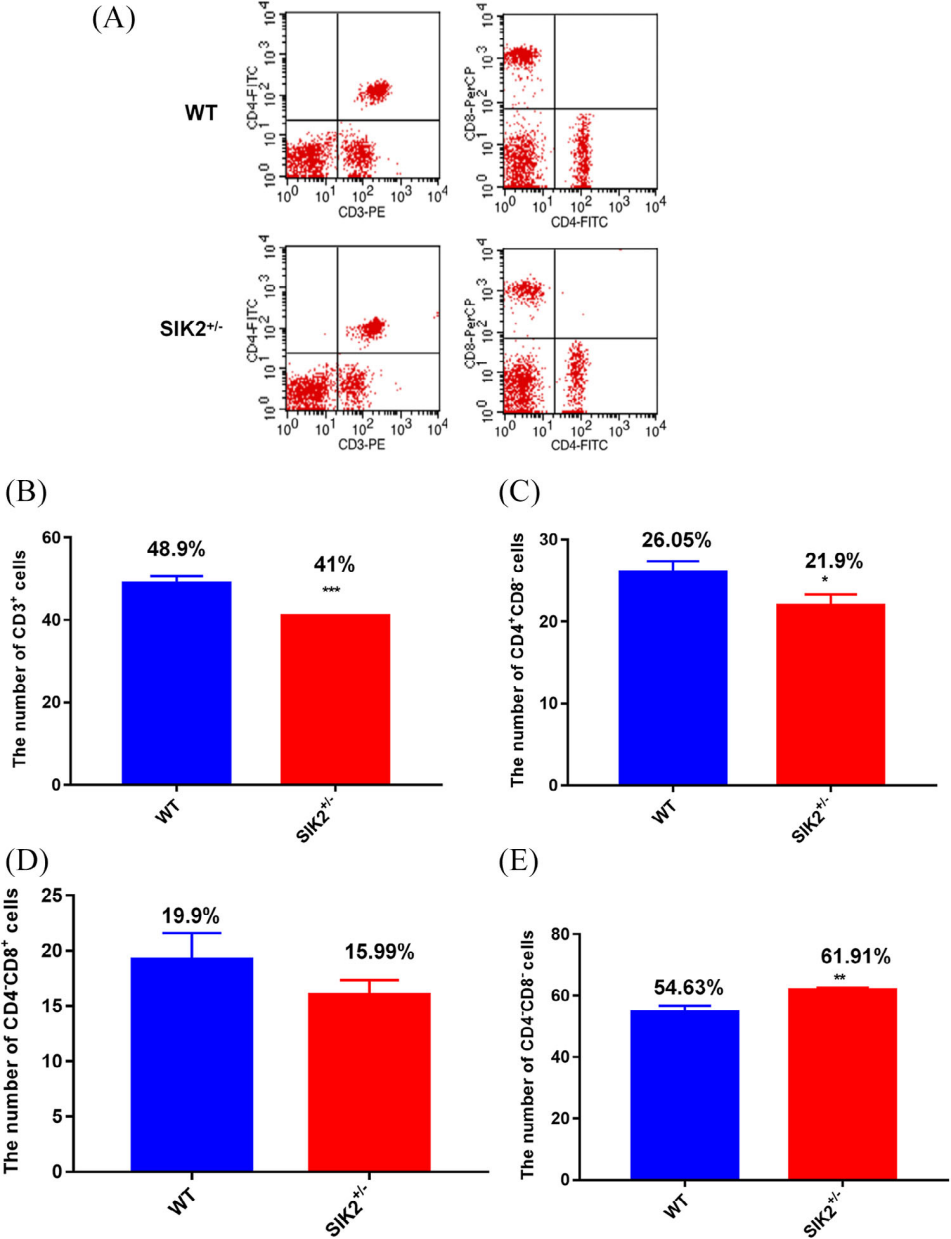
The deficiency of SIK2 reduces the number of maturation T cells in blood. (A) The flow cytometry analysis was performed in blood. (B) The number of CD3^+^ T cells of blood in WT and SIK2**^+/−^** mice. (C) The number of CD4^+^ CD8^−^ T cells of blood in WT and SIK2**^+/−^** mice. (D) The number of CD4^−^ CD8^+^ T cells of blood in WT and SIK2**^+/−^** mice. (E) The number of CD4^−^ CD8^−^ T cells of blood in WT and SIK2**^+/−^** mice. Data are mean as —x— ± *SD*, *p* values were determined by *t*‐test, and ^*^ *p* < 0.05, ^**^ *p* < 0.01 and ^**^ *p* < 0.001 mean the statistical difference significantly.

It has been reported that IR can cause immune function injury and the decrease of WBC associate with the immune function, indicating the deficiency of SIK2 could impair the immunity. To investigate whether SIK2 can regulate immunity injury, the flow cytometry analysis was performed in blood from WT and SIK2^+/–^ mice (Figure 4A). We counted the different marked cells and found that CD3^+^ T cells decreased from (Figure 4B), which means the total number decrease of T cells and SIK2 deficiency could induce immune injury. CD4^+^CD8^−^ T cells, a mature T cells (Figure 4C) significantly decreased in the blood from SIK2^+/−^ mice, and CD4^−^CD8^+^ T cells (Figure 4D) show a decrease trend in the blood from SIK2^+/−^ mice. However, the immature T cells characterized by CD4^−^CD8^−^ increased in the blood from SIK2^+/−^ mice (Figure 4E), suggesting the deficiency of SIK2 impaired maturation of T cells.

**FIGURE 5:**
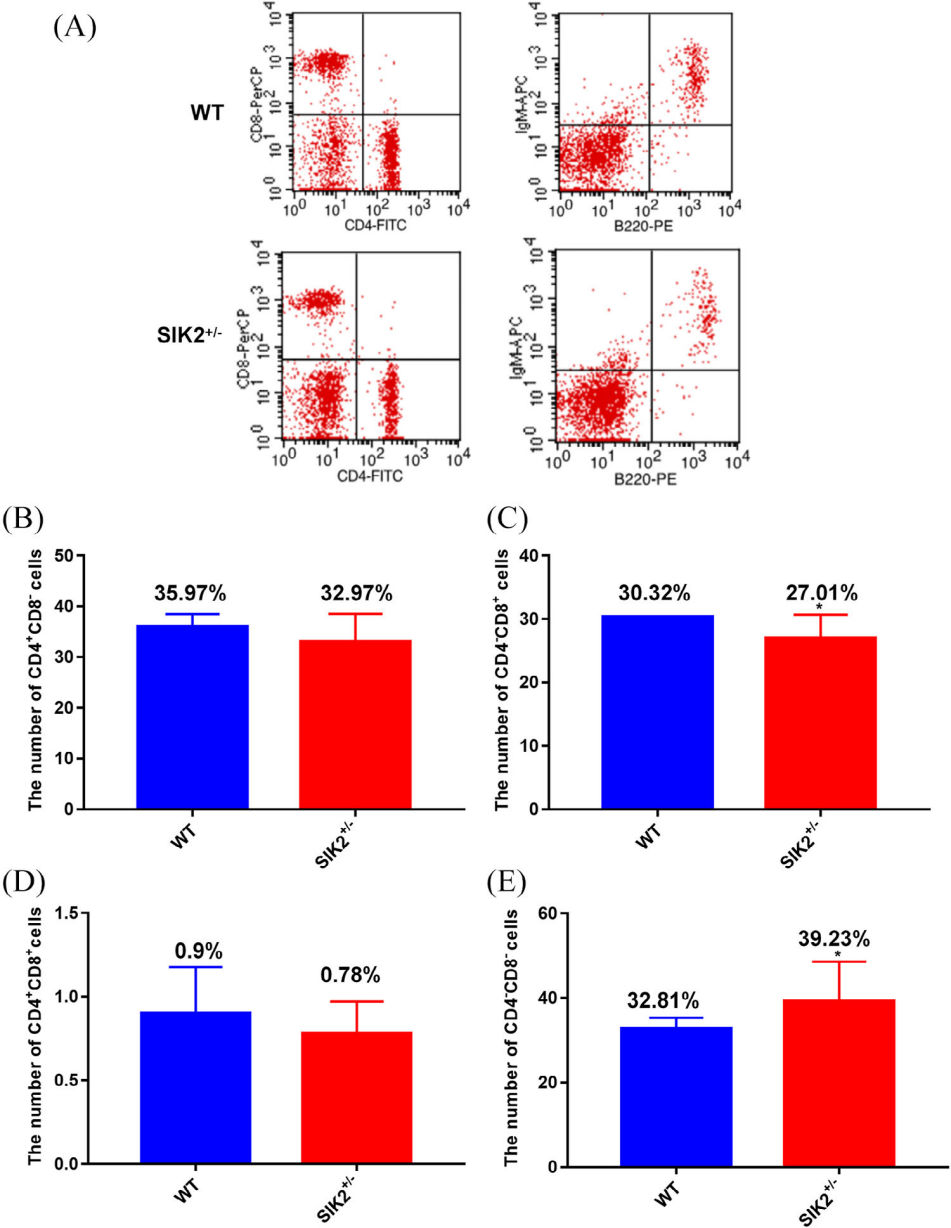
The deficiency of SIK2 inhibits the maturation of T cells in spleen. (A) The flow cytometry analysis was performed in spleen. (B) The number of CD4^+^ CD8^−^ T cells of spleen in WT and SIK2**^+/−^** mice. (C) The number of CD4^−^ CD8^+^ T cells of spleen in WT and SIK2**^+/−^** mice. (D) The number of CD4^+^ CD8^+^ T cells of spleen in WT and SIK2**^+/−^** mice. (E) The number of CD4^−^ CD8^−^ T cells of spleen in WT and SIK2**^+/−^** mice. Data are mean as —x— ± SD, *p* values were determined by *t*‐test, and ^*^ *p* < 0.05 mean the statistical difference significantly.

**FIGURE 6:**
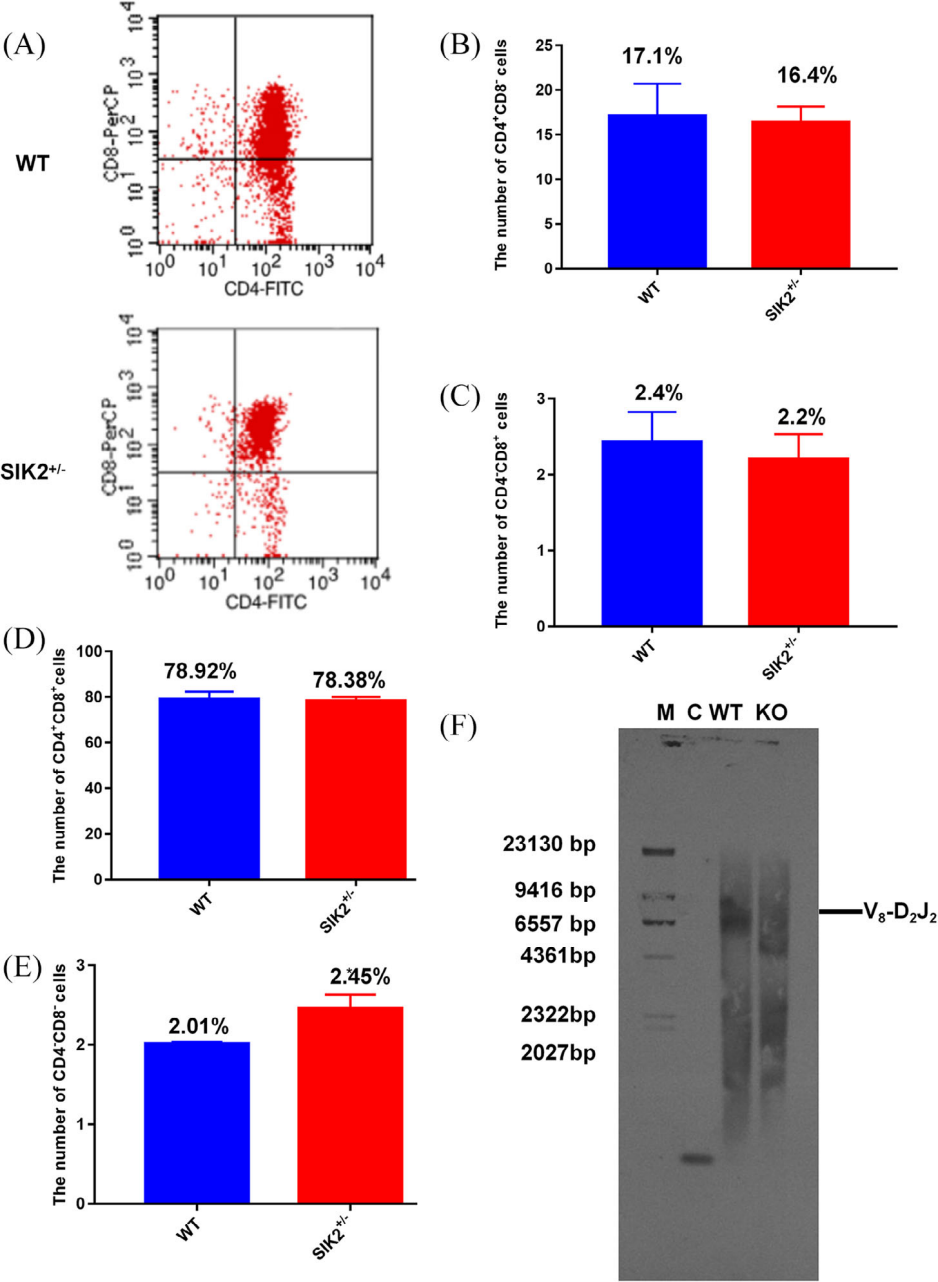
The deficiency of SIK2 inhibits the maturation of T cells in thymus. (A) The flow cytometry analysis was performed in thymus. (B) The number of CD4^+^ CD8^−^ T cells of thymus in WT and SIK2**^+/−^** mice. (C) The number of CD4^−^ CD8^+^ T cells of thymus in WT and SIK2**^+/−^** mice. (D) The number of CD4^+^ CD8^+^ T cells of thymus in WT and SIK2**^+/−^** mice. (E) The number of CD4^−^ CD8^−^ T cells of thymus in WT and SIK2**^+/−^** mice. (F) Southern blot to analyze the rearrangement of T‐cell receptor (TCR). Data are mean as —x— ± *SD*, *p* values were determined by *t*‐test, and ^*^ *p* < 0.05 mean the statistical difference significantly.

Spleen and thymus are the main source of T cells in peripheral blood, and considering the established critical role of spleen and thymus in immune regulation, The number of mature T cells, CD4+CD8‐T cells in spleen and thymus, did not change significantly after the deficiency of SIK2 (Figures 5A and B and 6A and B). The CD4^−^CD8^+^ T cells in spleen significantly reduced (Figure 5C); however, there was no significant decrease in the thymus of SIK2^+/−^ mice (Figure 6C). These data indicated that the deficiency of SIK2 might mainly impair function of the spleen. What is more, the immature T cells characterized by CD4^+^CD8^+^ in the spleen and thymus of SIK2^+/−^ mice showed no remarkable increase (Figures 5D and 6D). But the immature T cells characterized by CD4^−^CD8^−^ in spleen and thymus increased of SIK2^+/−^ mice (Figures 5E and 6E), which further suggested the deficiency of SIK2 impaired maturation of T cells. T‐cell receptor (TCR) can interact with MHC I (major histocompatibility complex I) or MHC II and participate in the differentiation and maturation process of T cells. The southern blot analysis demonstrated that the recombinant fragment of Vβ8‐Dβ2Jβ2 was hybridized at about 6557 bp in WT mice, while that fragment was hardly detected in SIK2^+/–^ mice (Figure 6F). These data indicated that the deficiency of SIK2 blocks the maturation of T cells.

**FIGURE 7:**
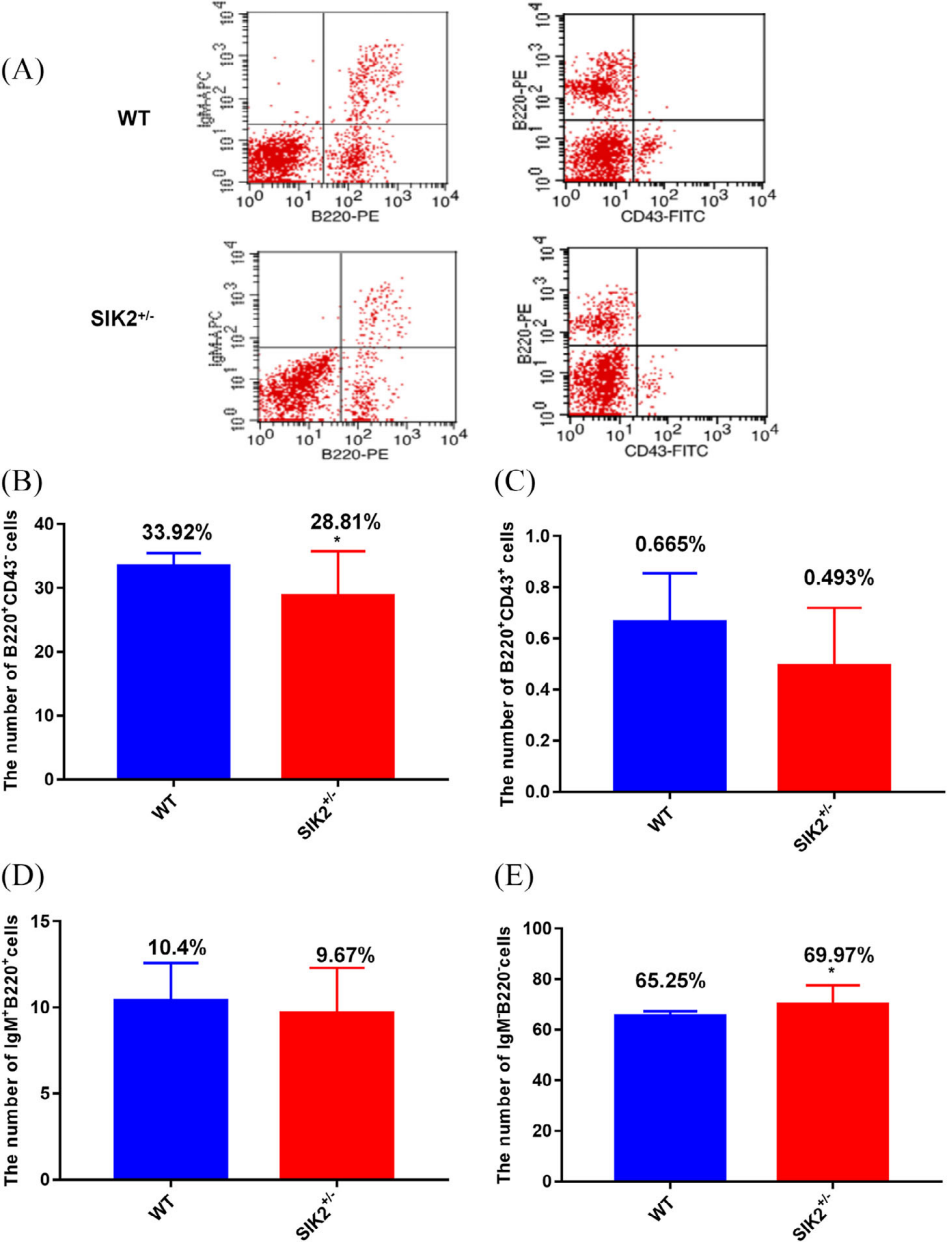
The deficiency of SIK2 reduces the number of maturation B cells in bone marrow. (A) The flow cytometry analysis was performed in bone marrow. (B) The number of B220^+^ CD43^−^ B cells of bone marrow in WT and SIK2**^+/−^** mice. (C) The number of B220^+^ CD43^+^ B cells of bone marrow in WT and SIK2**^+/−^** mice. (D) The number of IgM^+^ B220^+^ B cells of bone marrow in WT and SIK2**^+/−^** mice. (E) The number of IgM^−^ B220^−^ B cells of bone marrow in WT and SIK2**^+/−^** mice. Data are mean as —x— ± *SD*, *p* values were determined by *t*‐test, and ^*^ *p* < 0.05 mean the statistical difference significantly.

Bone marrow is the main source of B lymphocytes, which mainly provides humoral immunity. The flow cytometry analysis of cells from bone marrow suggested that B220^+^CD43^−^ B cells significantly reduced in SIK2^+/–^ mice and the B220^+^CD43^+^ and IgM^+^B220^+^ B cells had a no significantly change in bone marrow (Figure 7A–D). Conversely, the IgM^−^B220^−^ immature B cells in bone marrow increased (Figure 7E), indicating the deficiency of SIK2 blocks the maturation of B cells. Together, these data indicated that SIK2 is required for repair of immune injury.

## 3 DISCUSSION

SIK2 has important regulatory roles in glucose and lipid metabolism, melanin synthesis, neuronal survival, cell cycle and autophagy.^16^, ^18^, ^19^, ^20^, ^21^ In our previous study, we identified SIK2 interacts with DNA‐PKcs, and DNA‐PKcs is a critical protein for IR response. Our research demonstrated that the protein content of SIK2 significantly increased in RAW264.7 and AHH1 cells after IR treatment. RAW264.7 cells are commonly used to study immune injury is that they can be easily cultured and manipulated in vitro, which allows for controlled experimental conditions and reproducible results. Moreover, RAW264.7 cells express many of the same cell surface markers and cytokines as primary macrophages. This makes them a useful tool for investigating the molecular mechanisms underlying immune injury and for testing potential therapeutic interventions. However, RAW264.7 cells are a cell line and may not fully recapitulate the complexity of the immune system in vivo. Therefore, we have conducted the SIK2 knock out of mice by Cre‐LoxP system. Finally, we revealed that the deficiency of SIK2 can promote immune injury by inhibiting the maturation of lymphocytes.

Gene knockout technology is an essential tool to study gene function in line with the development of scientific research, which is of great significance for the development of life science and medicine. The Cre‐LoxP system was conducted in this research to establish the SIK2^+/–^ mice. At the beginning of this study, we aimed to acquire the SIK2^−/–^ mice. Although the process of constructing knockout mice was carefully planned and strictly controlled, and there was no SIK2 knockout mice was finally obtained. Many studies have been reported that SIK2 fully knockout mice can be obtained, and the SIK2 fully knockout mice can be obtained in other mouse strains, rather than C57BL/6. Therefore, analyzing the number of mice with different genotypes after mating of SIK2^+/–^ mice showed that the ratio of SIK2^+/+^ to SIK2^+/–^ was approximately 1 : 2 (Table S1). What is more, we took embryos at different time points after pregnancy, and found that SIK2^−/−^ embryos existed in 15 days post‐pregnancy and SIK2^−/−^ embryos were digested and absorbed at 16 days post‐pregnancy (Figure S3). Combining Mendel's genetic law and the function of SIK2, these results indicated that the SIK2^−/−^ mice died in an embryonic phase. Furthermore, detecting the knockout efficiency of SIK2 in mice heart, spleen, thymus, and lymph node showed that it has successfully decreased the protein expression of SIK2. Thus, all the results were conducted in SIK2^+/–^ mice and the SIK2^+/+^ as the control. And this also suggests that in later studies, the role and mechanism of SIK2 in radiation‐induced immune injury and the role of SIK2 in other radioactive tissue damage can be further studied through immune system specific knockout of SIK2.

It has been demonstrated that SIK3 is a target of regulating innate immunity.^2^, ^13^ Furthermore the absence of SIK2/3 can inhibit the progression to mature single positive T cells.^31^ However, the independent effects of SIK2 on immune injury and the maturation of T cells and B cells were not explored before. We observed the weight of body, thymus, and spleen significantly decreased in the SIK2 deficiency mice. Importantly, after the deficiency of SIK2, the number of mature T cells and B cells in blood, thymus, and bone marrow decreased, and the immature T cells and B cells in blood, thymus, and bone marrow increased. Moreover, the deficiency of SIK2 suppressed the rearrangement of the TCRβ. When the recombination of VDJ fails, the V region cannot be bind to the C region, and the complete TCRαβ chain cannot be formed, resulting in the blocked maturation of T cells.^32^ These data indicated that the deficiency of SIK2 has an effect on the maturation of T cells and B cells, that deficiency can be involved in immune injury. However, the molecular mechanism of SIK2 involvement in radiation‐induced immune injury is still worth further discussion. Based on the characteristics of SIK2 radiation responsiveness, we are currently further studying the role of SIK2 in other radioactive tissue damage.

In summary, our results indicate that the deficiency of SIK2 promoted immune injury by inhibiting the maturation of T cells and B cells. In addition, SIK2 is a responder of immune cells in IR. This study demonstrated the functions of SIK2 in vivo by constructing SIK2^+/–^ mice, laying a foundation for further study of SIK2 protein function, and providing a potential target for the treatment of cancer radiotherapy, immune dysfunction and other diseases.

## 4 MATERIALS AND METHODS

### 4.1 Cells culture and treatment

Mouse macrophage cells RAW264.7 were cultured in DMEM medium supplemented with 10% fetal bovine serum and 1% penicillin/streptomycin, AHH1 cells were cultured in RPMI1640 medium supplemented with 10% fetal bovine serum and 1% penicillin/streptomycin, and mainly cultured in a humidified incubator with 5% CO_2_ at 37°C. The ^60^γ‐ray (Beijing Institute of Radiation Medicine) was used to irradiate the cells at a dose rate of 66.63 cGy/min at room temperature.

### 4.2 Experimental animals

EIIα Cre^+^/^+^ homozygous mice were purchased from Cyagen (Guangzhou) Biosciences Inc. Transgenic, wild‐type, and heterozygous mice were purchased from the laboratory animal center of the Academy of Military Medical Sciences, and all the mouse were performed according to the animal care and use Committee of the Academy of Military Medical Sciences and the Laboratory Animal Guideline of Welfare and Ethics of China. Mice were maintained in openly ventilated cages with group housing (5 per cage), in a temperature‐controlled (20–26°C) and humidity‐controlled (50%−60%) facility with 12 h light/12 h dark cycle. These mice were maintained on a C57BL/6 background. And the target vector of SIK2 conditional knockout mice (pSIK2‐KO) was constructed by this laboratory.

### 4.3 Construction of SIK2 knockout mice

We built vector‐targeting carriers with two LoxP sites to transfect ES cells, and by homologous recombination, two LoxP loci were integrated into the genome located on introns on both sides of exon 2 and exon 3 of SIK2 gene. According to the recognition characteristics of the Cre enzyme, the sequence between the two LoxP sites will be effectively removed after its expression, leading to the deletion of exon 2 and 3. Finally, that deletion resulting in a frameshift mutation of SIK2 gene and producing a truncated protein containing 53 amino acids to achieve the purpose of gene knockdown. The detailed operation procedure is shown in Figure S4.

### 4.4 Western blot analysis

Total proteins of cells or tissues were extracted with M‐PER® Mammalian Protein Extract Regent (Thermo, USA) or T‐PER® Tissue Protein Extraction Reagent (Thermo, USA) on ice. And the protein concentration was determined by diquinoline formic acid method (BCA). Samples were separated by SDS‐PAGE and detected with indicating antibodies. The antibodies were used following the manufacturer's instructions, including SIK2 (D28G3, Cell Signaling Technology, USA, 1:1000), GAPDH (ZSGB‐Bio, Beijing, 1:1000), and β‐actin (ZSGB‐Bio, Beijing, 1:1000).

### 4.5 DNA isolation and PCR analysis

By cutting 5 cm mouse tail, genomic DNA was isolated and obtained. Add 80 μL of A solution (A solution 100 mL: 0.5 mL NaOH (5 M NaOH mother liquor), 40 μL EDTA (0.5 M, PH8.0)) to the tail, heat it at 95°C for 40 min, and oscillate every 10 min; Add 80 μL B solution (B solution 100 mL: 4 mL Tris‐HCl (1 M, PH8.0)) and shake well. After centrifugation at 3500 rpm for 10 min, the template DNA can be used for PCR analysis. All PCR primers for genotype identification and screening are in Table S2. The products of PCR amplification were subjected to 1% agarose gel electrophoresis, and the gel was exposed to UV light to visualize the target bands. In addition, the remaining PCR products were sent to sequencing (AuGCT, Beijing) to confirm whether the SIK2 gene was successfully knocked out.

### 4.6 Southern blot analysis

Genomic DNA from cells and tissues sample was extracted. The digested DNA was extracted with equal volumes of phenol and chloroform. Then resulting fragments were separated by 0.7% agarose gel electrophoresis (25 V, overnight at low temperature), blotted onto a nylon membrane, and hybridized with the specific probe for the Vβ8‐Dβ2Jβ2 recombinant fragment at the TCRβ site after prehybridization for 2 h. Subsequently, the membrane was blocked in blocking solution for 30 min, incubated in diluted antibody (Dig‐AP, 1:5000) for at least 30 min, then washing the membrane twice with buffer for 15 min each time. After dropping CSPD on the membrane for 5 min (isolated from air, 15−25°C), remove the excess liquid and incubate at 37°C for 10 min. Finally, develop with X‐ray film in the darkroom and record the results.

### 4.7 Whole blood cell count

Twenty microliters of blood from each mice were collected and analyzed using an animal hematology analyzer (TC20, BIORAD, USA). Whole blood cell count indicators included red blood cell count (RBC), platelet count (PLT), white blood cell count (WBC), and hemoglobin (HB or Hgb).

### 4.8 Flow cytometry

Cells were collected from peripheral blood, thymus, spleen, and bone marrow and washed with ice PBS. The cell concentration was adjusted to 1 × 106 cells/mL and added 100 μL of mouse cell suspension to the bottom of the flow tube. For analysis of the number of T cells, the antibody of CD4 (eBioscience, USA) and CD8 (eBioscience, USA) was used according to the manufacturer's instruction. For analysis of the number of B cells, the antibody of IgM (eBioscience, USA), B220 (eBioscience, USA), and CD43 (eBioscience, USA) was used according to the manufacturer's instruction. Fluorescent labeled antibodies for cell surface staining at 4°C and react for 40 min in the dark. Two milliliters of PBS were added and centrifuged at 300 × *g* for 5 min to wash the cells, adding 0.5 mL PBS to resuspend cells. Finally, fluorescence‐activated cell sorting (FACS, BD, USA) analysis was performed by flow cytometry.

### 4.9 Detection of TCRβ gene rearrangement

Thymocyte genomic DNA was isolated from SIK2^+/+^ (WT) and SIK2^+/−^ (KO) mice, using SNB112‐F/R as probe primers to synthesize specific probes for the Vβ8‐Dβ2Jβ2 recombinant fragments at the TCRβ site. Southern blot analysis was used to detect whether there is a difference in the V(D)J recombination at the TCRβ site between WT and KO mice. PCR was used to prepare probe templates; the cycling conditions for genomic DNA were as follows: predenaturation at 95°C for 5 min, followed by 35 cycles of 95°C for 30 s, 58°C for 30 s, 72°C for 30 s.

### 4.10 Statistical analysis

Student's unpaired *t*‐test for comparison of means was used to compare between two groups with SPSS 20.0 software. A *p* value less than 0.05 was considered to be statistically significant. Values of ^*^ *p* < 0.05, ^**^ *p* < 0.01, and ^***^ *p* < 0.001 were considered as statistically significant.

## AUTHOR CONTRIBUTIONS

**Jiaojiao Zhu**: data curation; writing the manuscript. **Chao Li**: performing the experiments; data analysis. **Ping Wang**: performing the experiments; writing‐original draft. **Yuhao Liu**: assisting with the experiment. **Zhongqiu Li**: assisting with the experiment. **Zhongmin Chen**: critical regents. **Ying Zhang**: critical regents. **Bin Wang**: critical regents. **Xueping Li**: assisting with the experiment. **Ziyan Yan**: assisting with the experiment. **Xinxin Liang**: assisting with the experiment. **Shenghui Zhou**: assisting with the experiment. **Xingkun Ao**: assisting with the experiment. **Maoxiang Zhu**: study concept. **Pingkun Zhou**: study concept; supervision. **Yongqing Gu**: study concept; critical design; supervision; validation. All authors have read and approved the final manuscript.

## CONFLICT OF INTERESTS STATEMENT

The authors declare that there is no conflict of interest regarding the publication of this article.

## ETHICS STATEMENT

All animal procedures were approved by Animal Laboratory of Laboratory animal center Academy of Military Medical Sciences (IACUC‐DWZX‐2021‐761). All methods were performed in accordance with the relevant guidelines and regulations. All animal experiments met the ARRIVE guidelines.

## Supporting information

Supporting Information

## Data Availability

The original contributions presented in the study are included in the article, and further inquiries can be directed to the corresponding author.
