# Supplementary material for: Deficiency of salt‐inducible kinase 2 (SIK2) promotes immune injury by inhibiting the maturation of lymphocytes
Source: MedComm (2020). 2023 Sep 11;4(5):e366. doi: 10.1002/mco2.366 (PMC10495731; doi:10.1002/mco2.366)
Supplement: Supplementary file 1 — Supporting Information [file MCO2-4-e366-s001.docx]

**Deficiency of SIK2 Promotes Immune Injury by Inhibiting the Maturation of Lymphocytes**

**Running title: Function of SIK2 in radiation induced immune injury**

Jiaojiao Zhu^1#^, Chao Li^2#^, Ping Wang^1#^, Yuhao Liu^1^, Zhongqiu Li^3^, Zhongmin Chen^4^, Ying Zhang^3^, Bin Wang^2^, Xueping Li^2^, Ziyan Yan^1^, Xinxin Liang^5^, Shenghui Zhou^5^, Xingkun Ao^5^, Maoxiang Zhu^1*^, Pingkun Zhou^1*^ and Yongqing Gu^1,3,5*^

^1^ Beijing Key Laboratory for Radiobiology, Beijing Institute of Radiation Medicine, Beijing 100850, P. R. China;

^2^ School of Life Science, Shihezi University, Shihezi, Xinjiang Province832003, P. R. China;

^3^ Medical School, Shihezi University, Shihezi, Xinjiang Province 832003, P. R. China

^4^ PLA Rocket Force Characteristic Medical Center, Beijing100088, P. R. China;

^5^ Hengyang Medical College, University of South China, Hengyang, Hunan Province 421001, P. R. China.

# Jiaojiao Zhu, Chao Li and Ping Wang contributed equally to this work

*Correspondence should be addressed to Yongqing Gu, [yqgu96@163.com](mailto:yqgu96@163.com); Pingkun Zhou, [zhoupk@nic.bmi.ac.cn](mailto:zhoupk@nic.bmi.ac.cn) and Maoxiang Zhu, [zhumx@nic.bmi.ac.cn](mailto:zhumx@nic.bmi.ac.cn)

**Table S1. PCR primers.**

| Primer ID | Sequence (5’-3’) |
| --- | --- |
| LoxP_F: | GAAGTCTCTCTGTGTATTATTTGGACCC |
| LoxP_R: | TGCTTGTTCATGCAACATACATATCTT |
| Neo-Del F: | GGGAGAAACTTGCCACCTTACAAGAG |
| Neo-Del R: | GGTCAGGCAAGGCAAAACAGAAG |
| Cre-GT-F1: | CTCTAGCGTTCGAACGCACTGA |
| Cre-GT-R1: | GGATCATCAGCTACACCAGAGACG |
| Exon3-F: | TGTGGTGGCTCAGCTAGACTACAGCC |
| Exon3-R: | TGGCAAGTTTCTCCCTATCTTTTGCTATC |
| NGH001- R1: | GCTAAATGCCTGAAAGCAACCTATT |
| NGH001- F1: | GAAGTCTCTCTGTGTATTATTTGGACC |
| NGH001_3’PCR_F: | GCTGACCGCTTCCTCGTGCTTTA |
| NGH001_3’PCR_R: | GAACACTATATGGTCTGCTGCCCAACTA |
| Neo Probe-F: | CTTGCTCCTGCCGAGAAAGT |
| Neo Probe-R: | GGCGATACCGTAAAGCACGA |
| NGH001_5’PCR_F: | GTGCATGCTATAGCACTGTGGCACAT |
| NGH001_5’PCR_R: | TACGAAGTTATACGTGTCGACGTGCAC |
| SNB112-F | CAGGCAGGTCCAGTGAG |
| SNB112-R | GGAGACATTGTACCCCTTAG |

**TableS2 Number of mice with different genotypes**

| Genotype | number |
| --- | --- |
| SIK2^+/+^ | 49 |
| SIK2^+/-^ | 108 |
| SIK2^-/-^ | ns |

**SIK2^+/+^: Wild type group; SIK2^+/-^: the heterozygous deficiency of SIK2 group; SIK2^-/-^: the**

**homozygous deficiency of SIK2.**


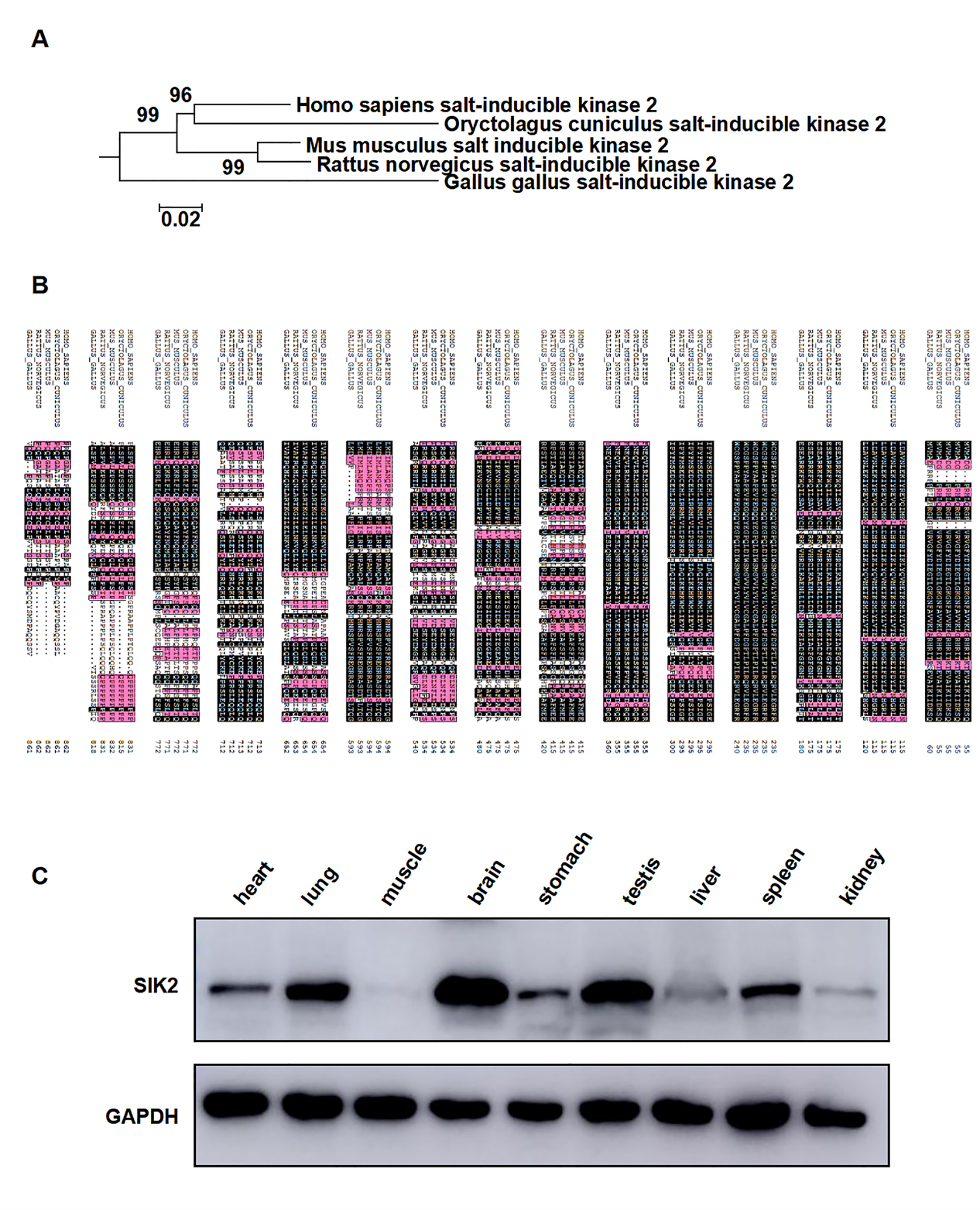


**Figure S1 SIK2 is highly conserved during biological evolution.**

1. Bioinformatic analysis of SIK2 gene in human, Oryctolagus cuniculus, Mus musculus, Rattus norvegicus and Gallus gallus sequences was performed by MEGA version 6.0 software. **B.** The SIK2 amino acid sequences of that five typical species were aligned using the DNAMAN software. C.The expression profile of SIK2 protein.


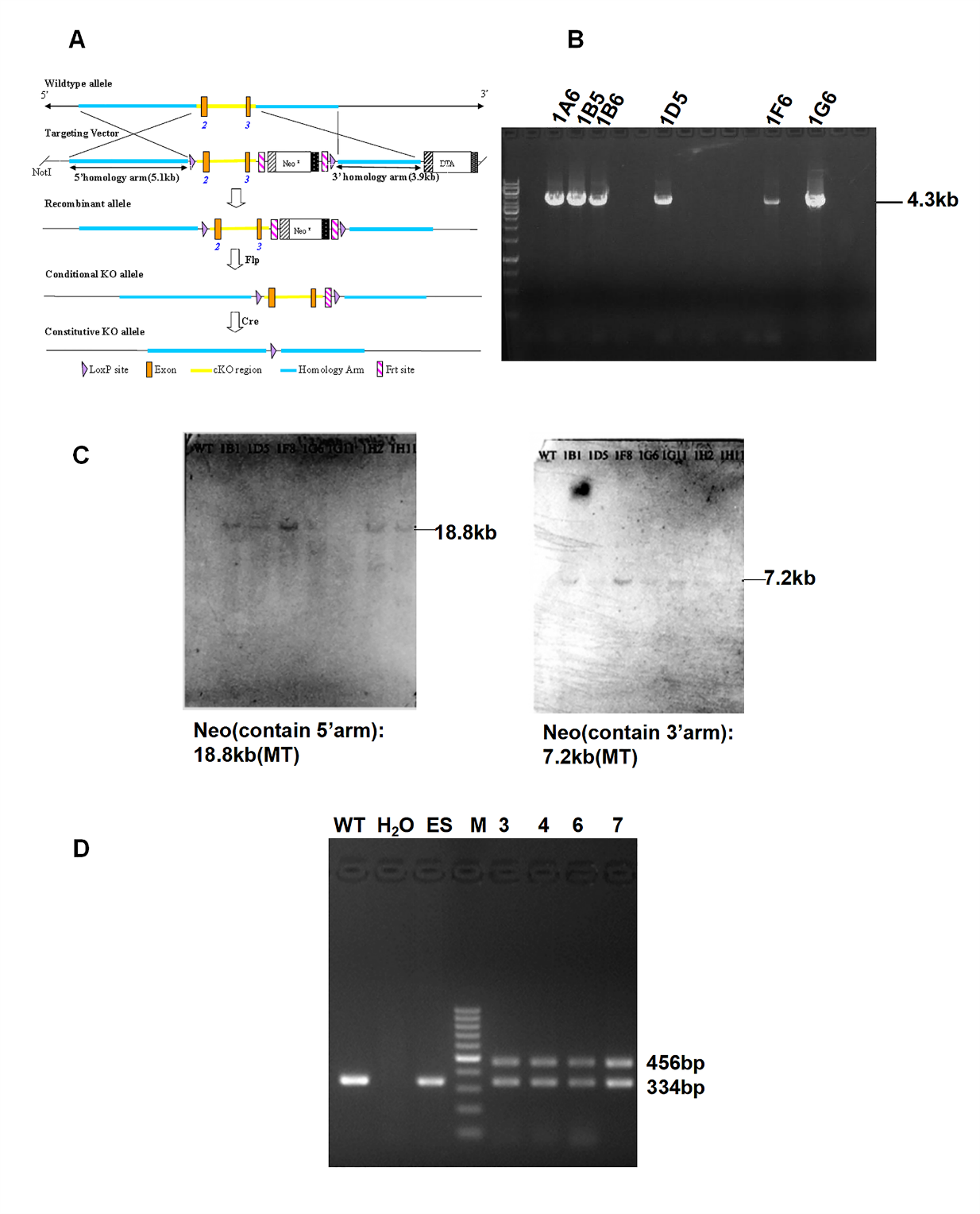


**Figure S2 The construction of SIK2^+/-^ mice.**

**A.** The knockout process and target site. **B.** PCR to identify whether homologous recombination was successfully performed. **C.** Southern Blot to identify the ES-positive clones. **D.** PCR to identify the Neo locus was successfully removed.


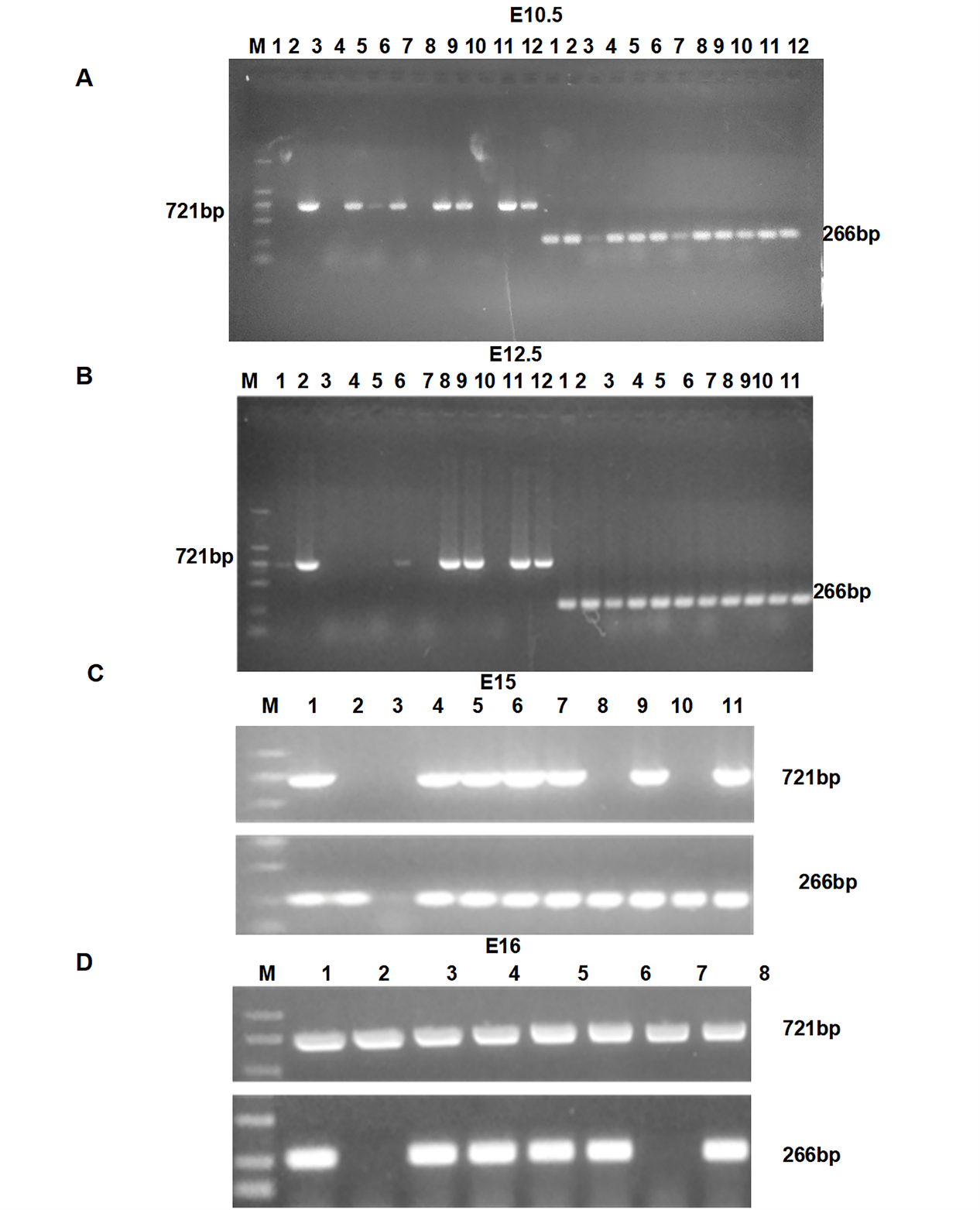


**Figure S3 The identification of SIK2^+/-^ mice.**

1. Genotype identification of embryos on day 10.5. **B.** Genotype identification of embryos on day 12. **C.** Genotype identification of embryos on day 15. **D.** Genotype identification of embryos on day 16.


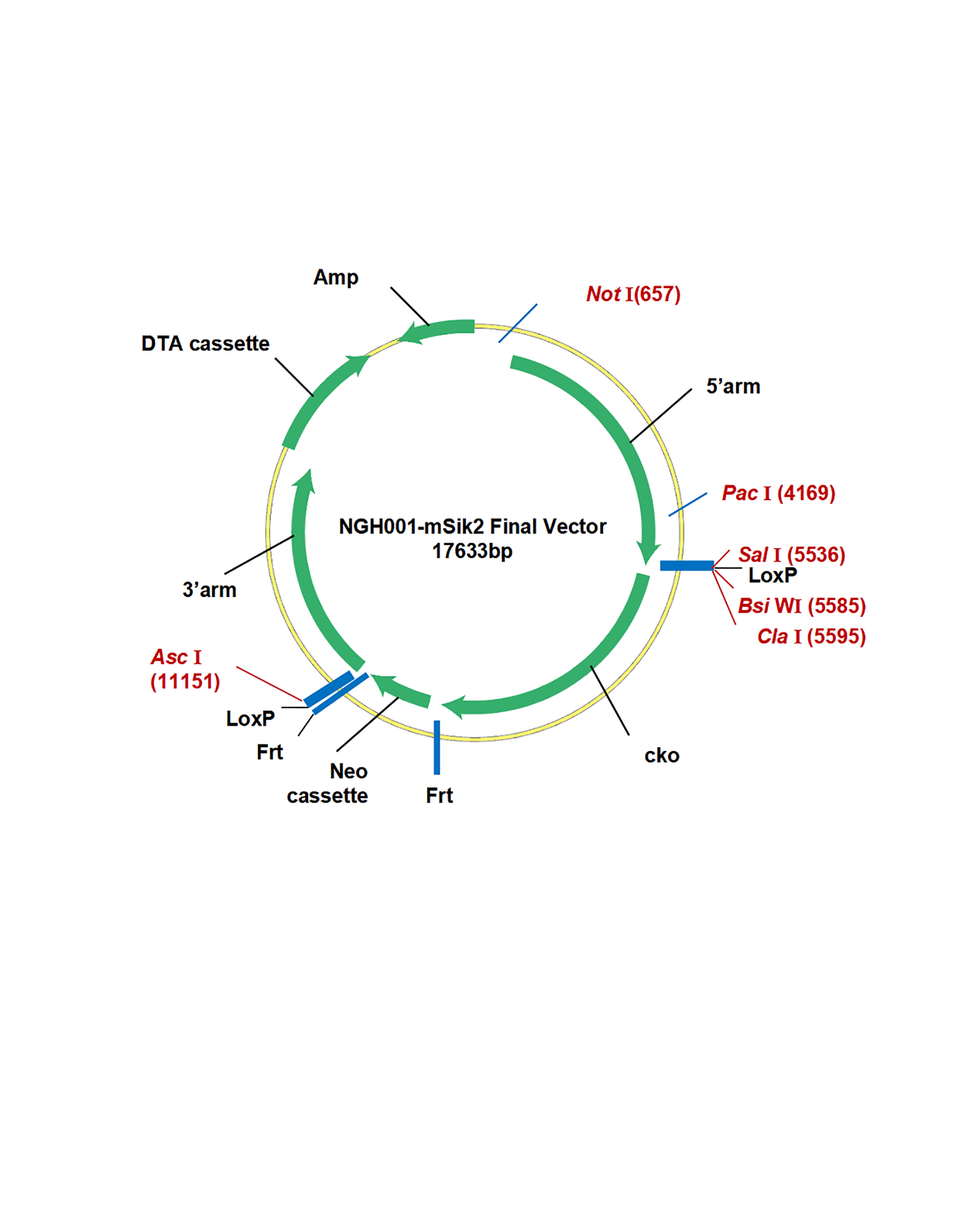


**Figure S4 The construction of target vector in SIK2 conditional knockout mice.**
